# Supplementary material for: Phytochemical Investigation of New Algerian Lichen Species: Physcia Mediterranea Nimis
Source: Molecules. 2021 Feb 20;26(4):1121. doi: 10.3390/molecules26041121 (PMC7924039; doi:10.3390/molecules26041121)
Supplement: Supplementary file 1 [file molecules-26-01121-s001.zip › Figure S2.docx]

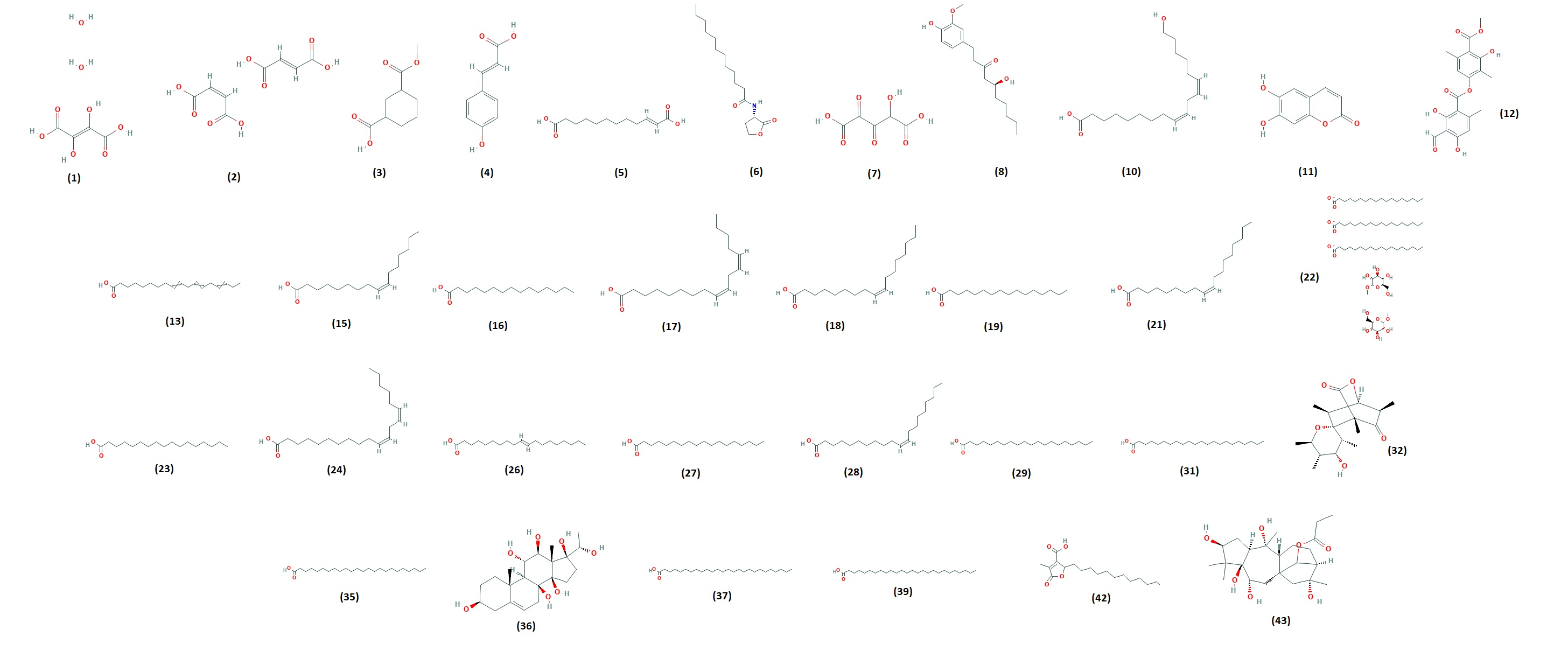


**Figure S2.** Chemical structure of Compounds identified in in *WA-hex* of *Physcia mediterranea* by UHPLC/ESI/MS/MS.
