# Supplementary material for: Phytochemical Investigation of New Algerian Lichen Species: Physcia Mediterranea Nimis
Source: Molecules. 2021 Feb 20;26(4):1121. doi: 10.3390/molecules26041121 (PMC7924039; doi:10.3390/molecules26041121)
Supplement: Supplementary file 1 [file molecules-26-01121-s001.zip › Figure S4.docx]

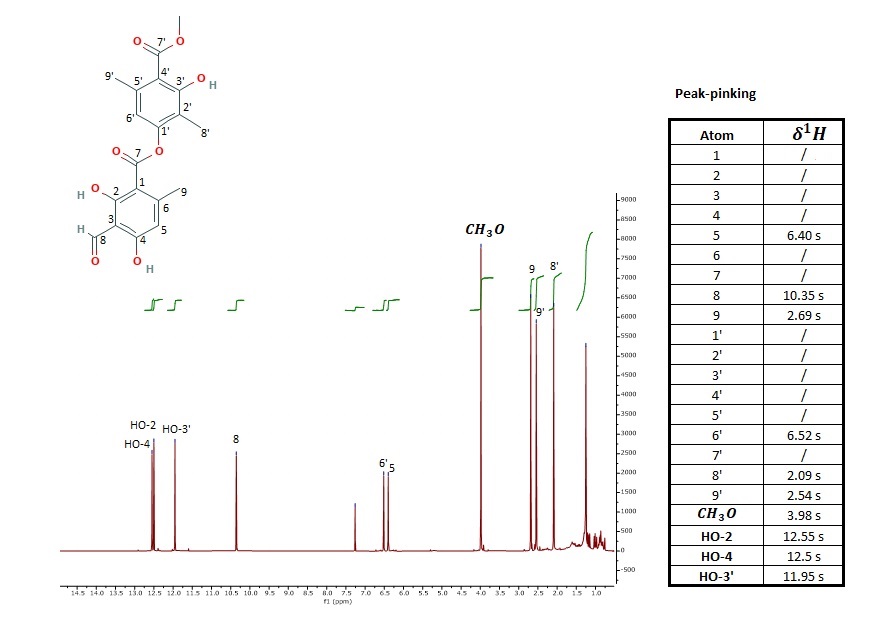


**Figure S4.** 1H NMR spectrum of WA-hex (400 MHz). Chemical shift values are standardised to the residual CDCL3 signal at 7.26 ppm. The multiplicity of signals is given as follows: s, singlet; d, doublet; q, quadruplet.
