# Supplementary material for: Phytochemical Investigation of New Algerian Lichen Species: Physcia Mediterranea Nimis
Source: Molecules. 2021 Feb 20;26(4):1121. doi: 10.3390/molecules26041121 (PMC7924039; doi:10.3390/molecules26041121)
Supplement: Supplementary file 1 [file molecules-26-01121-s001.zip › Figure S6.docx]

**
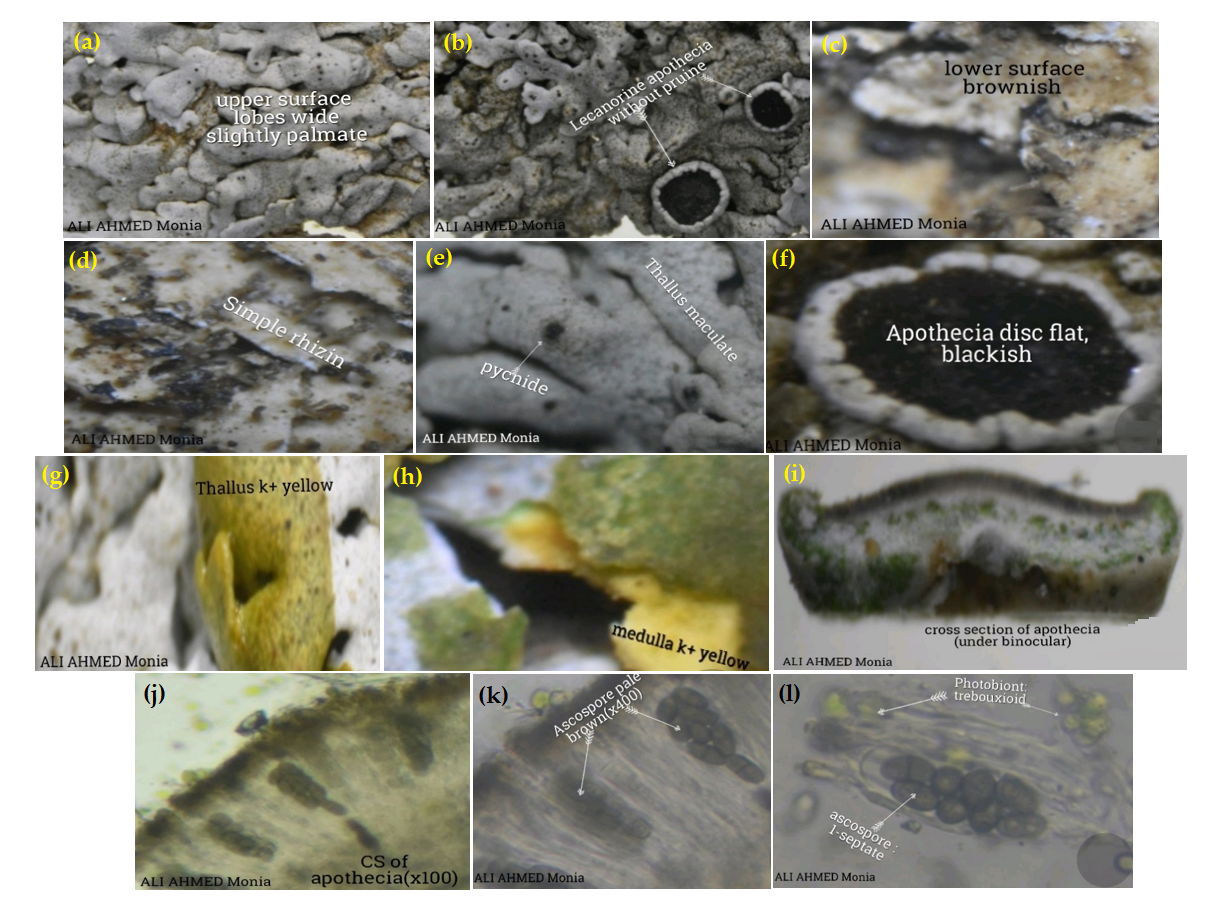
**

**Figure S6**: *Physcia mediterranea* Nimis: Binocular observations(a,b,c,d,e,f,g,h,i) and histological sections under microscope (j,k,l).

**Description of *Physcia mediterranea* Nimis**

**S**yn: *Physcia aipolia* f. *saxicola* = *Physcia aipolia* subsp. *scopulorum* = *Physcia scopulorum*

Thallus: foliose with very elongated lobes, convex, pale grey, clearly visible macules, no soralia, no isidia.

Photosymbiont: green algae (genus Trebouxia).

Chemistry: thallus and medulla K + yellow.

Apothecia: lecanorin, black disc, crenellated thallin margin, 1-septum brown spores.
