# Supplementary figures and images for: Phytochemical Investigation of New Algerian Lichen Species: Physcia Mediterranea Nimis
Source: Molecules. 2021 Feb 20;26(4):1121. doi: 10.3390/molecules26041121 (PMC7924039; doi:10.3390/molecules26041121)

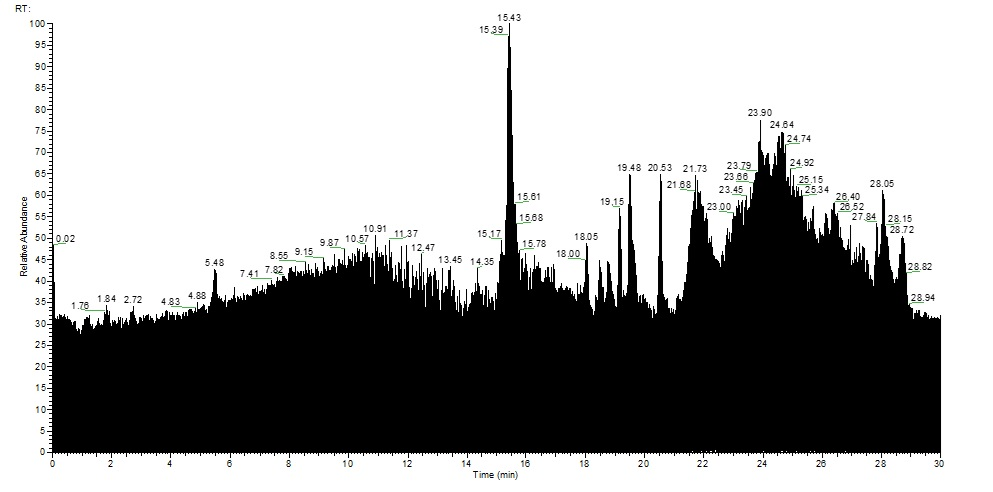


**Figure S1.** UHPLC/ESI/MS/MS chromatogram of *Physcia mediterranea* (*WA-hex*).

Supplement: Supplementary file 1 [file molecules-26-01121-s001.zip › Figure S1.docx]

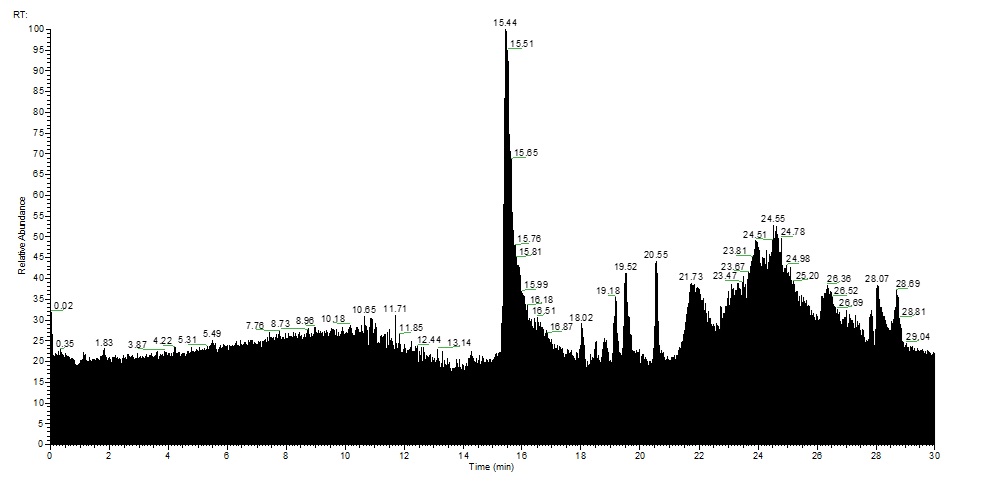


**Figure S3.** UHPLC/ESI/MS/MS chromatogram of *Physcia mediterranea (*ME*-hex*).

Supplement: Supplementary file 1 [file molecules-26-01121-s001.zip › Figure S3.docx]
